# Intimate partner violence during pregnancy and its association with birth asphyxia in hospitals of Tigray region, Ethiopia

**DOI:** 10.1186/s12887-024-04585-6

**Published:** 2024-02-13

**Authors:** Kahsay Zenebe Gebreslasie, Gelawdiwos Gebre, Dawit Zenebe, Rahel Nardos, Aklil Birhane

**Affiliations:** 1 College of Health Sciences, Mekelle University, Mekelle, Ethiopia; 2 Oregon Health& Science University, USA Portland,

**Keywords:** Intimate partner violence, Pregnancy, Birth asphyxia

## Abstract

**Background:**

Birth asphyxia is the main cause of neonatal mortality and morbidity worldwide. Some studies indicate intimate partner violence during pregnancy is a risk factor for birth asphyxia. In Ethiopia, intimate partner violence during pregnancy is reported to be high. Despite this high prevalence, there is a lack of data about the association of birth asphyxia and intimate partner violence. The aim of this study was to assess the prevalence of intimate partner violence during pregnancy and its associated factors with birth asphyxia in health facilities in the Tigray region of northern Ethiopia.

**Methods:**

This was an institutional-based cross-sectional study conducted at select health facilities in the Tigray region of Ethiopia. Random sampling technique was employed to select health facilities and systematic sampling was used to select 648 study participants. Data was entered by using Epi info version 3.5.1 and was analyzed using SPSS version 20. Bivariate and multivariate analysis was done to assess the association between exposure to intimate partner violence during pregnancy and birth asphyxia after adjusting for possible confounders.

**Results:**

The prevalence of intimate partner violence during pregnancy was 47(7.3%). Eighty two (12.7%) babies were delivered with birth asphyxia. Intimate partner violence during pregnancy had a significant association with birth asphyxia, AOR (95% CI) = 4.4(2-9.8). In addition to this, other factors that were associated with birth asphyxia include place of residence [ AOR (95% CI) = 2.7(1.55–4.8)], age > 19 [AOR (95% CI) = 2.9(1.29–6.5)], age 20–35 [AOR (95% CI) = 3.1(1.06–9.3)], gestational age < 37 weeks [AOR(95% CI) = 7.2(3.5–14.8)] and low birth weight [AOR(95% CI) = 3.9(2.1–7.3)].

**Conclusions:**

The prevalence of birth asphyxia in this study is high and is further increased by intimate partner violence during pregnancy. Health care providers and policy makers should take measures aimed at preventing intimate partner violence during pregnancy to reduce harm to the mother and adverse birth outcomes.

## Background

Intimate partner violence (IPV) is among the most common violence against women and it is remains a major public health and human rights issue [1, 2]. Based on data from a multicounty study, About 71% of women experience, physical, sexual or both types of violence in their lifetime [3]. The occurrence of IPV among pregnant mothers ranges from 2% in Australia to 13.5% in Uganda [4]. A systematic review from African countries showed that, IPV during pregnancy ranged from 2 to 57% [5] Studies from Ethiopia demographic health survey and from eastern region of Ethiopia showed that the prevalence of IPV among pregnant women were 28.74% [6] and 39.81% [7] respectively. Researchers have shown that IPV during pregnancy has negative impact on the health of the mothers’ and their children [2].

APGAR score, a means of evaluating newborn’s immediate condition after birth, is low in asphyxiated baby and is highly correlated with neonatal mortality [8]. Birth asphyxiated babies had 34.5 times higher risk of mortality compared to non-birth asphyxiated babies [9]. Study from Ethiopia shown that magnitude of birth asphyxia was 19.3 [10]. Similarly, study conducted in Tigray the magnitude of birth asphyxia was 22.1% [11]. According to the Ethiopian Demographic Health Survey (EDHS) of 2016, there was a perinatal mortality rate is 33 per 1000 pregnancies [12]. The survey also indicated that neonatal mortality was 95 per 1000 live births [12].

Studies show that birth asphyxia is related to severe disability such as low cognitive function, neurological problem and subtle cognitive impairment [13, 14]. Although some studies demonstrated an association between IPV during pregnancy and birth asphyxia [15], and that IPV during pregnancy is associated with the need for neonatal intensive care [16]. On the other hand, some studies reported no association between IPV and birth asphyxia [17]. The differences in findings might be due to regional variability and underreporting of IPV. In Ethiopia, especially in the Tigray region, there is little if any research done to investigate the association between IPV during pregnancy and birth asphyxia.

Although Ethiopia has formulated different policies to avert the rate of neonatal mortality and morbidity, it still remains unacceptably high. Given the high prevalence of IPV in Ethiopia, it is critical to understand the potential role of IPV on the high rate of neonatal mortality and morbidity. Given that IPV is potentially amenable to intervention, this research will inform policy makers and health professionals and can have important implications on neonatal outcomes.

## Methods

### Study design, area and period

Institution-based cross-sectional study design was used. The study was conducted from from February-June 2018 among women who gave birth in Tigray Regional hospital. Tigray region is located 783 km from Addis Ababa, the capital city of Ethiopia. Based on the 2007 census, the population was estimated to be 4,316,988. Women of child bearing age (15–49) comprise 251,650 of the population. According to the 2015 Tigray Regional Health Bureau annual report, there were a total of one specialized hospital, 15 general hospitals, 22 primary hospitals, 204 health centers, and 712 health posts that were federally run. There were also three private hospitals. According to Mini EDHS 2019, 48% of women gave birth at a health facility [18].

### Study population

Women who gave birth in selected hospitals of Tigray region and who were present during the data collection time were included in the study. Babies with visible congenital anomalies and still birth were excluded from the study.

### Sample size determination and sampling technique

To calculate the sample size, we used available data that indicates 25.8 percent prevalence of IPV during pregnancy in Ethiopia [19] 21 and 95% confidence interval, 5% margin of error, design effect 2 and expected non-response rate 10%. Based on this, the calculated sample size was 648 and we calculated birth asphyxia by considering the magnitude of birth asphyxia from Gondar 13.8% [20], 95% confidence interval, 5% margin of error, design effect 2 and expected non-response rate 10%. Based on this, the calculated sample size was 403 which less than from 648. There are 41 hospitals (1 specialized hospital, 15 general hospitals, 22 primary hospitals, and 3 private hospitals) which provide delivery services in the study area. Health facilities were stratified into private and public hospitals, we are considering as heterogonous and sample size was allocated proportional based on their client follow. For this study, one private hospital (kalkidan) and eight public hospitals(Mekelle general Hospital, Ayder specilazed hospital, Shire general hospital, Adwa general hospital, Axum general hospital, Wukro general hospital, Abidy adi general hospital and Samre primary hospital) were selected by a simple random sampling technique. Participants from each health facility were selected by systematic sampling. Every 3rd postpartum woman was included until the required sample size was reached. If the selected participant was not eligible, then the next participant was included. The average client load for each facility during the 3 months preceding data collection was used as a basis for proportional allocation to each health facility and to find the interval.

### Data collection tools and processes

A questionnaire were prepared first in English, then translated into Tigrigna, and again back translated to English by native language experts to keep the consistency of the questionnaires. Data on maternal demographics, socioeconomic status, IPV and obstetrics factors was collected during discharge time by trained interviewers using pretested questionnaires. Low birth weight was assigned if the neonate weighed < 2500 g, and preterm birth was assigned if the neonate was born at < 37 co[mpleted weeks of gestation but > 28 weeks.

To maintain data accuracy of birth asphyxia, we trained data collectors and senior midwifes they used a standardized data collection tool, with data collected starting from the onset of labor and ending five minutes postpartum. Birth asphyxia was evaluated by based on the apgar score. Apgar score was assessed by using five variables. 1) Breathing effort: regular breathing score 2; irregular breathing and less than 30 breath/minute, score 1; non 0. 2) Regularity of heart rate: 100 beats/minute or more, score 2; less than 100 beats/minute score 1; none score 0. 3) Movement and muscle tone: active score 2; moderate score 1; limp score 0. 4) Skin color: pink score 2; bluish extremist score 1; totally bluish 0. 5) reflex response to stimuli: crying score 2; whimpering score 1; silence 0. All values were summed by the investigator and values below seven were considered as birth asphyxia.

To measure IPV during pregnancy, participants were asked if they have a history of physical violence during pregnancy such as being slapped, pushed or shoved, hit with a fist or something else that could hurt, beaten on her abdomen, choked or burnt on purpose, or being attacked or threatened by a knife, guns or other weapons. Participants were also asked about a history of emotional violence during pregnancy such as being insulted, humiliated, intimidated on purpose, and threatened. Sexual violence was defined as being forced to have sexual intercourse without consent, complying to have sexual intercourse due to fear, or being forced to do something sexual that she found degrading or humiliating. If a participant was found to have been exposed to one or more of the above violence types, she was identified as having been exposed to IPV during pregnancy [21].

The data was collected by nine midwives (diploma level training) and supervised by four midwives (bachelor level training).To maintain data quality, the questionnaire was pretested on 10% of the total sample size. Training was given for both data collectors and supervisors about the aim of the study, procedures, and how to approach the study participants and data collection techniques. Data was checked daily by the supervisors and investigators for completeness. Since IPV is a very sensitive issue for participants, interviews were made in a private room to maintain the confidentiality of the interviewees and to encourage them to speak up.

### Statistical analysis

Double data entry was done using EPI Info version 3.4.1, 2008 and the data was exported to SPSS version 20 software package for analysis.

To determine the association between maternal exposure to intimate partner violence and birth asphyxia, logistic regression analyses were done, and odds ratios with 95% confidence intervals were calculated. Multivariable logistic regression analysis was performed where intimate partner violence plus other variables that could affect the newborn were included. The degree of association between independent and dependent variables were assessed using an odds ratio with 95% confidence interval and significant association was considered at *P* value of < 0.05.

## Results

### Socio-demographic characteristics

A total of 647 participants took part in this study with a response rate of 99.8%. Out of the total respondents, 458 (70.78%) were urban residents. The mean age of the respondents was 27 ± 6 years. Majority of the respondents 530(81.9%) were between ages 20 – 35 years old. Of the total participants, 610(94.28%) were married and 301 (46.5%) were housewives (Table 1).

**Table 1:** Socio-Demographic characteristics of respondents, Tigray, North, Ethiopia, 2018

Variable		Frequency	Percent
Residence	Urban	458	70.78
	Rural	189	29.22
Age	≤ 19	50	7.7
	20–34	530	82
	≥ 35	67	10.3
Religion	Orthodox	581	89.8
	Muslim	66	10.2
Educational Status	Unable to read &write	108	16.7
	Read and write	44	6.8
	Primary education	175	27
	Secondary education and college	211	32.6
	Diploma and above	109	16.8
Marital Status	Married	610	94.3
	Single	37	5.7
Occupational status	Housewife	301	46.5
	Merchant	71	11
	Farmer	127	19.6
	Private employee	42	6.5
	Governmental employee	95	14.7
	Others	11	1.7

### Obstetrical characteristics of the participants

Nearly a quarter (24%) of the women delivered by cesarean section. Out of the 647, 162 (25%) women had premature rupture of the membrane (PROM), 66(10.2%) had hypertension during their pregnancy, and 35 (5.4%) had antepartum bleeding. Out of all the women who gave birth in the the study hospitals, 611(94.4%) had at least one ANC follow-up for their last baby. Forty two (6.5%) postpartum women had an unwanted pregnancies. In addition, 70 (10.8%) women gave birth before term and 120 (18.5%) of the babies had low birth weight. The prevalence of birth asphyxia in this study was 12.7% (82) (Table 2).

**Table 2:** Obstetrics characteristics of respondents, Tigray, North, Ethiopia, 2018

Variable		Frequency	Percentage
Mode of delivery	Vaginal	492	76
	C/S	155	24
PROM	Yes	162	25
	No	485	75
Hypertension	Yes	66	10.2
	No	581	89.8
APH	Yes	35	5.4
	No	612	94.6
ANC follow up	Yes	611	94.4
	No	36	5.6
Pregnancy wanted	Yes	605	93.5
	No	42	6.5
Preterm delivery	Yes	70	10.8
	No	577	89.2
Low birth weight	Yes	120	18.5
	No	527	81.5

Of the total participants, 288 (44.5%) women admitted that they ingested alcohol during pregnancy sometimes and 10(1.6%) ingested chat (a stimulant leaf) while they were pregnant with the index neonate. Three (0.5%) women had a habit of smoking while they were pregnant. Among partners (husbands), 19(2.9%) drank alcohol always, 362 (56%) drank alcohol sometimes, 31(4.8%) ingested chat sometimes, 4 (0.6%) ingested chat always, 22 (3.4%) smoked tobacco sometimes and 3(0.5) smoked tobacco always.

### Prevalence of intimate partner violence

Of the total study participants, 47 (7.3%) experienced intimate partner violence during their most recent pregnancy. Of these, 22 (46.8%) experienced physical violence, 8(17%) psychological violence, and 39 (60%) sexual violence.

### Association between intimate partner violence during pregnancy and birth asphyxia

Factors with *p* value ≤ 0.2 in the bivariate regression were IPV, rural residence, younger age at pregnancy, gestational age less than 37 weeks, ANC follow up, APH and low birth weight. These factors were exported to the multivariate regression. In the multivariate regression, IPV, rural residence, younger age at pregnancy, gestational age less than 37 weeks and low birth weight had significant associations (*p* < 0.05).

Women who experienced intimate partner violence during pregnancy have 4.4 times more likely to develop the risk of birth asphyxia compared to their counterparts (AOR (95% CI) = 4.4(2–9.8). Women from rural areas have 2.7 times increased risk of birth asphyxia compared to women from urban areas (AOR = 2.7(95%CI, 1.55–4.8). Women who are < 19 years old have 2.9 times increased risk of birth asphyxia compared to women who are 35 years old (AOR = 2.9(95%CI,1.29–6.5). Women with ages between 20–35 have 3.1 times increased risk of birth asphyxia compared to women with ages greater than 35 (AOR = 3.1(95%CI,1.06–9.3). In the same way, gestational age less than 37 weeks have 7.2 times increased risk of birth asphyxia (AOR = 7.2(95CI,3.5–14.8)and low birth weight have 3.9 times increased risk of birth asphyxia compared to counterpart (AOR = 3.9(95% CI, 2.1–7.3) (Table 3).

**Table 3:** Bivariate and multivariable logistic regression analyses of birth asphyxia by socio demographic variables, health-related variables, and intimate partner violence during pregnancy

**Variables**		**Birth asphyxia**		**COR(95% CI)**	**AOR(95%CI)**
		**Yes**	**No**		
Marital status	Marriage	77	533	1:00	
	Single	5	32	1.08(0.4–2.8)	
Resident	Urban	41	417	1:00	1:00
	Rural	41	148	2.8(1.75–4.5)	2.7(1.55–4.8)
Religion	Orthodox	74	507	1:00	
	Muslim	8	58	.94(.43–2)	
Age	< 19	13	37	2.8(1.4–5.5)	2.9(1.29–6.5)
	20–35	59	471	2(.79–5)	3.1(1.06–9.3)
	> 35	10	57	1:00	1:00
IPV	Yes	15	32	3.7(1.9–7.24)	4.4(2–9.8)
	No	67	533	1:00	1:00
Hypertension	Yes	10	56	1:00	
	No	72	509	.79(.38–1.6)	
APH	Yes	8	27	1:00	
	No	74	538	.46(.2–1.06)	1.3(.48–3.8)
Habit of alcohol intake	Never	44	315	1:00	
	Sometimes	38	250	1.08(.68–1.7)	
Pregnancy wanted	Yes	76	529	1:00	
	No	6	36	1.16(.47–2.8)	
ANC follow up	Yes	74	537	1:00	1:00
	No	8	28	2(.91–4.7)	1.33(.46–3.8)
Mode of delivery	Vaginal	64	428	1.1(.65–1.98)	
	Cesarean delivery	18	137	1:00	
Gestational age	< 37 week	35	35	11.2(6.4–19.6)	7.2(3.5–14.8)
	> = 37 week	47	530	1:00	1:00
Low birth weight	Yes	45	75	7.9(4.8–13)	3.9(2.1–7.3)
	No	37	490	1:00	1:00

## Discussion

This study which assessed the association between IPV during pregnancy and birth asphyxia provided new and important information that has been missing from research in low-income countries like Ethiopia. The magnitude of IPV during was 7.3% and it is associated with birth asphyxia. The magnitude of intimate partner violence during pregnancy is concerning and can have important implications on neonatal outcome. In this study, the prevalence was 7.3%. This is higher than studies conducted in high-income countries such as in Northeastern City reported 3.7% [22]. But it is lower than studies done in Vietnam (32.5%), Tanzania (30%), Ethiopia (Hosanna region) (23%), and southeast Ethiopia (25.8%) [17, 19, 23, 24]. The differences are likely partly due to true variations in prevalence, but could also be due to differences in study methodologies, timing of study, and under reporting of IPV. In Ethiopia, for example, the two studies done in Hosanna and southeast Ethiopia might be due to changes in partner violence awareness between 2013 and 2014.

In our study, the prevalence of birth asphyxia was 12.7%. A study done in the University of Gondar’s Hospital and in Jimma zone public hospital found similar findings with prevalence of 13.8% and 12.5% respectively [20, 25]. This study result is higher than in the study done in Dilchora Referral Hospital, in Dire Dawa city administration where the magnitude of birth asphyxia was 3.1% [26]. The difference might be due to differences in risk factors, study area characteristics, and the limitations of documentation intrinsic to retrospective data collection.

Our study found that IPV during pregnancy is significantly associated with asphyxia. This finding is in line with a research done among women in Massachusetts who were abused during pregnancy, and found to have an increased risk of birth asphyxia compared to those women not exposed to abuse during pregnancy [15]. Other study also supported that IPV during pregnancy associated with received neonatal intensive care [16]. Women who are exposed to IPV may experience more hypertension and reduced blood flow and oxygen to the fetus during labor [17]. Furthermore, physical violence may predispose women to placental abruption, which in turn may lead to fetal distress [21].

In this study, birth asphyxia has an association with place of residence, women's age, gestational age, and low birth weight. Women who come from rural areas have 2.7 times the risk of birth asphyxia compared to women from urban areas. This may be due to delayed access to timely obstetric care that disproportionately impacts women from rural areas compared to women from urban areas [27].

In our study, women who delivered before 37 weeks had about seven times higher risk of birth asphyxia compared to women with term pregnancy. This finding is similar to research done in Debremarkos where women who gave birth before 37 weeks had a higher risk of birth asphyxia [28]. Similarly, another research done in Nigist Eleni Mohammed memorial teaching hospital, showed that women who delivered before 37 weeks had an increased risk of birth asphyxia [29]. This is not surprising as babies born preterm have immature respiratory systems that predispose them to birth asphyxia [30].

Similarly, our findings show that babies born with low birth weight has four times increased risk of birth asphyxia compared to normal weight babies. This finding is supported by multiple other studies including the ones done in Beijing Obstetrics and Gynecology Hospital and sub Saharan African, [31, 32]. The pathophysiology for this is likely similar to that of preterm babies in which the respiratory systems are not fully developed [30].

The current study also revealed that women whose age is less than 35 years old were about 3 times more likely to have birth asphyxia compared to those women whose age is greater than 35 years old. This finding is supported by research conducted in Dilchora Referral Hospital, in Dire Dawa ‘where women’s age less than 25 years were risk factors for birth asphyxia [26]. This might be due to the fact that most women greater than 35 years old are multipara with shorter duration of labor, thus reducing the risk of birth asphyxia.

### Strength of the study

The strength of this study is that it is a large, prospective, well-sampled cross-sectional study that includes both rural and urban participants. It is also the first study to look at the association between IPV and birth asphyxia in the Tigray region of Ethiopia.

### Limitations of the study

The main limitation of this study is that we cannot infer direct causal relationships between IPV and birth asphyxia.

### Conclusions and recommendations

This study showed that the incidence of birth asphyxia is highly associated with intimate partner violence during pregnancy. This finding emphasizes the critical need for providing IPV screening during pregnancy and the importance of providing much needed counseling and Preventive services to women. Community awareness should include men and community leaders who can play a significant role in reducing IPV during pregnancy. Organizations working to restore gender equity should align their missions to health care workers with one of their goals being reducing IPV during pregnancy.

## Data Availability

The data used in this study are available from the corresponding author and possible to share upon reasonable request.

## Abbreviations

IPV: Intimate partner violence
PROM: Premature rapture of membrane
ANC: Antenatal care
EDHS: Ethiopia demographic health survey
COD: Crude odds ratio
AOR: Adjusted odds ratio
